# Synthesis and protective effect of new ligustrazine-vanillic acid derivatives against CoCl_2_-induced neurotoxicity in differentiated PC12 cells

**DOI:** 10.1186/s13065-017-0250-z

**Published:** 2017-02-28

**Authors:** Bing Xu, Xin Xu, Chenze Zhang, Yuzhong Zhang, GaoRong Wu, Mengmeng Yan, Menglu Jia, Tianxin Xie, Xiaohui Jia, Penglong Wang, Haimin Lei

**Affiliations:** 1 School of Chinese Pharmacy, Beijing University of Chinese Medicine, Beijing, 100102 China; 2 Department of Pathology, Beijing University of Chinese Medicine, Beijing, 100102 China

**Keywords:** T-VA amide derivatives, Neuroprotective effect, Synthesis, PC12 cell

## Abstract

Ligustrazine-vanillic acid derivatives had been reported to exhibit promising neuroprotective activities. In our continuous effort to develop new ligustrazine derivatives with neuroprotective effects, we attempted the synthesis of several ligustrazine-vanillic acid amide derivatives and screened their protective effect on the injured PC12 cells damaged by CoCl_2_. The results showed that most of the newly synthesized derivatives exhibited higher activity than ligustrazine, of which, compound **VA-06** displayed the highest potency with EC_50_ values of 17.39 ± 1.34 μM. Structure-activity relationships were briefly discussed.

**Graphical abstract:**
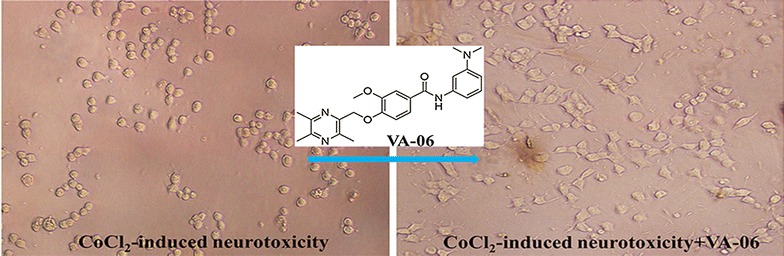
New series of ligustrazine-vanillic acid amide derivatives were synthesized and evaluated for their protective effect on the injured PC_12_ cells damaged by CoCl_2_. **VA-06** was found to be the most active one

New series of ligustrazine-vanillic acid amide derivatives were synthesized and evaluated for their protective effect on the injured PC_12_ cells damaged by CoCl_2_. **VA-06** was found to be the most active one

## Background

Ischemic stroke is one of the leading causes of death and disability in the world [1–3]. It is clear that even a brief ischemic stroke may trigger complex cellular events that ultimately lead to the neuronal cell death and loss of neuronal function [1, 4, 5]. Although remarkable progress has been made in treating stroke, effective approaches to recover damaged nerve are not yet to be found [6–9]. Therefore, it is necessary to develop new generation of neuroprotective agents with neural repair-promoting effect.

**Fig. 1:**
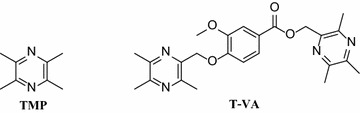
Structures of TMP and T-VA

Ligustrazine (tetramethylpyrazine, TMP) (Fig. 1) is a major effective component of the traditional Chinese medicine *Chuanxiong* (*Ligusticum chuanxiong hort*), which is currently widely used in clinic for the treatment of stroke in China. It has been reported to show beneficial effect on ischemic brain injury in animal experiments and in clinical practice [10–14].

Meanwhile previous studies showed that many of aromatic acids, such as vanillic acid, protocatechuic acid, salicylic acid, exhibited interesting neuroprotective activity [15–19]. In our previous effort to develop new neuroprotective lead compounds, inspired by the potent bioactivities of TMP and aromatic acids on neuroprotection, we designed and synthesized several series of ligustrazine derivatives by incorporation of ligustrazine with aromatic acids. The neuroprotective activity detection revealed that some compounds presented potent protective effects on injured differentiated PC12 cells, of which **T-VA** (3,5,6-trimethylpyrazin-2-yl)methyl3-methoxy-4-((3,5,6-trimethylpyrazin-2-yl)methoxy)benzoate) (Fig. 1) exhibited high potency with EC_50_ values of 4.249 µM [20–22]. Meanwhile, recent research has demonstrated that **T-VA** exerted neuroprotective in a rat model of ischemic stroke [23].

In continuation of our research, we decided to undertake a study of the ligustrazinyl amides, because amides relatively have metabolic stability when compared to ligustrazinyl esters [24]. In this study, we reported the design, synthesis of the novel T-VA amide analogues containing different types of amide fragments, as well as in vitro neuroprotective activities screening on the injured PC12 cells. And the structure-activity relationships (SARs) of these novel compounds were also briefly discussed.

## Results and discussion

### Chemistry

**Scheme 1:**
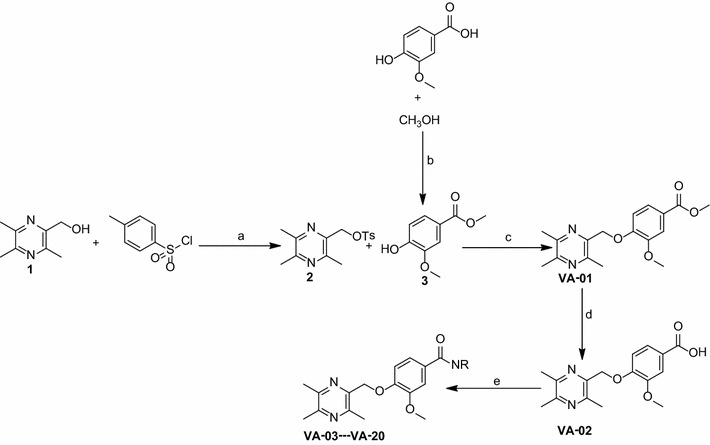
Synthesis of the ligustrazine-vanillic acid derivative **VA-01**–**VA-20**. Reagents and Conditions: **a** dry THF, KOH, 4-toluene sulfonyl chloride (Tscl), 25 °C, 15 h; **b** thionyl chloride (SOCl_2_), 25 °C, 15 h; **c** DMF, dry K_2_CO_3_, N_2_, 70 °C, 15 h; **d** THF:MeOH:H_2_O = 3:1:1, LiOH, 37 °C, 2 h; **e** DCM, HoBt, EDCI, DIPEA, 25 °C, 12 h

All the target compounds were synthesized via the routes outlined in Scheme 1. The key intermediate (3,5,6-trimethylpyrazin-2-yl)methanol (**1**) was prepared according to our previous study [25]. As shown in Scheme 1, compound **1** underwent sulfonylation reaction with 4-toluene sulfonyl chloride to afford the intermediate **2**. Starting from vanillic acid, the intermediate **3** was prepared by reacting vanillic acid with methyl alcohol and thionyl chloride. Then the intermediate **3** were reacted with the intermediate **2** in N,N-Dimethylformamide (DMF) in the presence of potassium carbonate to afford the compound **VA-01**, which was then hydrolyzed under alkaline conditions to give the target compound **VA-02**.

The derivatives **VA-03**–**VA-23** were successfully obtained by coupling **VA-02** with various amines in the presence of 1-[3-(dimethylamino) propyl]-3-ethyl-carbodiimide hydrochloride (EDCI), diisopropylethylamine (DIPEA) and 1-hydroxybenzotriazole (HOBt) in CH_2_Cl_2_. The structures of all the target compounds (Table 1) were confirmed by spectral (^1^H-NMR, ^13^C-NMR) analysis and high resolution mass spectrometry (HRMS).

**Table 1:** The structures of ligustrazine derivatives **VA-01**–**VA-20**

Compound	R	Yield (%)
**VA-01**	CH_3_O–	52.5
**VA-02**	OH–	98.1
**VA-03**	CH_3_CH_2_NH–	89.5
**VA-04**		65.2
**VA-05**	CH_3_NH–	87.0
**VA-06**		74.0
**VA-07**		68.9
**VA-08**		76.4
**VA-09**		86.7
**VA-10**		79.3
**VA-11**		68.3
**VA-12**		57.6
**VA-13**		65.7
**VA-14**		57.8
**VA-15**		68.9
**VA-16**		67.0
**VA-17**		65.2
**VA-18**		62.7
**VA-19**		75.1
**VA-20**		83.2

### Protective effect on injured PC12 Cells

Setting ligustrazine and **T-VA** as the positive control drug, the neuroprotective activity of target compounds was evaluated on the neuronal-like PC12 cells damaged by CoCl_2_. The results, expressed as proliferation rate (%) at different concentration and EC_50_, were summarized in Table 2. As shown in Table 2, most of the ligustrazine-vanillic acid amide derivatives showed better protective effects than the positive control drug **TMP** (EC_50_ = 64.35 ± 1.47 µM) on injured differentiated PC12 cells. Among the candidates, the compound **VA-06** exhibited the most potent neuroprotective activity with EC_50_ values of 17.39 ± 1.34 µM.

**Table 2:** The EC_50_ of the ligustrazine-vanillic acid amide derivatives for protecting damaged PC12 cells

Compd	Proliferation rate (%)					EC_50_ (μM)^a^
	60 μM	30 μM	15 μM	7.5 μM	3.75 μM	
**VA-01**	81.75 ± 2.34	49.05 ± 4.07	43.15 ± 3.11	21.25 ± 1.25	22.77 ± 7.27	18.74 ± 1.94
**VA-02**	7.38 ± 0.95	12.55 ± 1.50	−0.47 ± 1.97	−11.43 ± 2.05	−10.48 ± 1.68	>100
**VA-03**	25.50 ± 1.48	21.42 ± 1.35	18.63 ± 0.82	13.34 ± 1.68	7.36 ± 1.73	52.48 ± 2.0
**VA-04**	46.60 ± 2.14	40.99 ± 3.08	41.49 ± 2.89	23.64 ± 2.32	6.88 ± 1.89	29.61 ± 0.78
**VA-05**	37.17 ± 2.17	31.36 ± 3.78	25.65 ± 2.05	21.54 ± 2.19	17.11 ± 1.51	36.61 ± 1.97
**VA-06**	89.81 ± 3.02	51.80 ± 5.61	29.51 ± 4.15	17.32 ± 6.10	15.78 ± 3.01	17.39 ± 1.34
**VA-07**	8.79 ± 2.27	53.07 ± 2.41	47.15 ± 1.31	7.42 ± 1.00	−5.52 ± 2.14	60.20 ± 25.70
**VA-08**	52.64 ± 2.94	29.29 ± 2.93	23.41 ± 1.71	18.50 ± 3.61	26.69 ± 5.58	33.62 ± 3.96
**VA-09**	49.34 ± 1.80	41.80 ± 0.81	41.56 ± 1.51	23.14 ± 2.78	14.05 ± 3.78	27.90 ± 1.65
**VA-10**	16.33 ± 1.60	33.99 ± 2.61	12.56 ± 4.21	15.66 ± 4.06	15.60 ± 5.67	48.79 ± 3.76
**VA-11**	32.99 ± 2.82	23.38 ± 2.92	15.20 ± 2.54	11.09 ± 0.67	14.44 ± 4.85	47.85 ± 1.84
**VA-12**	−71.58 ± 2.70	−59.50 ± 3.91	−35.73 ± 3.44	−11.99 ± 4.56	13.86 ± 2.28	>100
**VA-13**	−277.39 ± 4.12	−292.67 ± 10.71	−297.34 ± 12.0	−298.64 ± 8.39	−296.33 ± 11.32	>100
**VA-14**	15.86 ± 1.47	12.13 ± 1.17	8.64 ± 0.83	5.51 ± 0.69	2.69 ± 0.72	71.66 ± 2.12
**VA-15**	−198.39 ± 4.52	−60.74 ± 3.21	88.57 ± 7.11	48.83 ± 5.28	45.01 ± 8.01	>100
**VA-16**	−23.15 ± 3.05	−13.96 ± 1.49	−14.86 ± 2.64	−14.51 ± 1.40	2.99 ± 1.08	>100
**VA-17**	69.41 ± 4.00	52.29 ± 3.05	32.78 ± 0.96	18.63 ± 0.81	10.12 ± 0.59	24.73 ± 1.37
**VA-18**	5.32 ± 1.11	12.04 ± 0.44	15.96 ± 1.05	15.27 ± 0.74	−2.97 ± 0.85	71.92 ± 1.07
**VA-19**	15.21 ± 3.12	13.89 ± 2.96	8.23 ± 1.31	8.61 ± 1.45	10.52 ± 2.03	65.72 ± 2.93
**VA-20**	25.14 ± 4.22	17.38 ± 0.21	15.87 ± 1.05	15.12 ± 0.65	8.97 ± 0.49	53.74 ± 1.69
**TMP**	14.44 ± 0.76	12.24 ± 0.66	11.82 ± 0.45	10.80 ± 0.43	9.65 ± 0.71	64.35 ± 1.47
**T-VA**	127.27 ± 3.70	118.60 ± 7.47	88.59 ± 2.28	51.49 ± 1.14	31.01 ± 0.94	4.29 ± 0.47

^a^Mean value ± standard deviation from three independent experiments

From the obtained results, it was observed that esterification at the carboxylic group of vanillic acid may contribute to enhance the neuroprotective activity, such as **VA-01** > **VA-02**. This was in agreement with our previous research [20]. It should be noticed that introduction of a large lipophilic aromatic amine residue leaded to complete loss of neuroprotective activity (with exception of **VA-06**), such as **VA-13**–**VA-16**. But the compounds that introduced an aromatic amine residue at the carboxylic group of vanillic acid performed better neuroprotective activities than **VA-02** without any group substituted, such as **VA-03**, **VA-04**, **VA-05**, **VA-08** > **VA-02.** Furthermore, the structure-activity relationship analysis among the **T-VA** aromatic amide derivatives revealed that the neuroprotective activities were mainly influenced by the type, but not the alkyl chain length of amine substituents, as exemplify by **VA-04** > **VA-03**, **VA-05**. Although none of the newly synthesized T-VA derivatives showed more effect than the positive control drug **T-VA**, the structure-activity relationship (SAR) analysis above provided important information for further design of new neuroprotective ligustrazine derivatives.

### Protective effect of VA-06 on injured PC12 cells

**Fig. 2:**
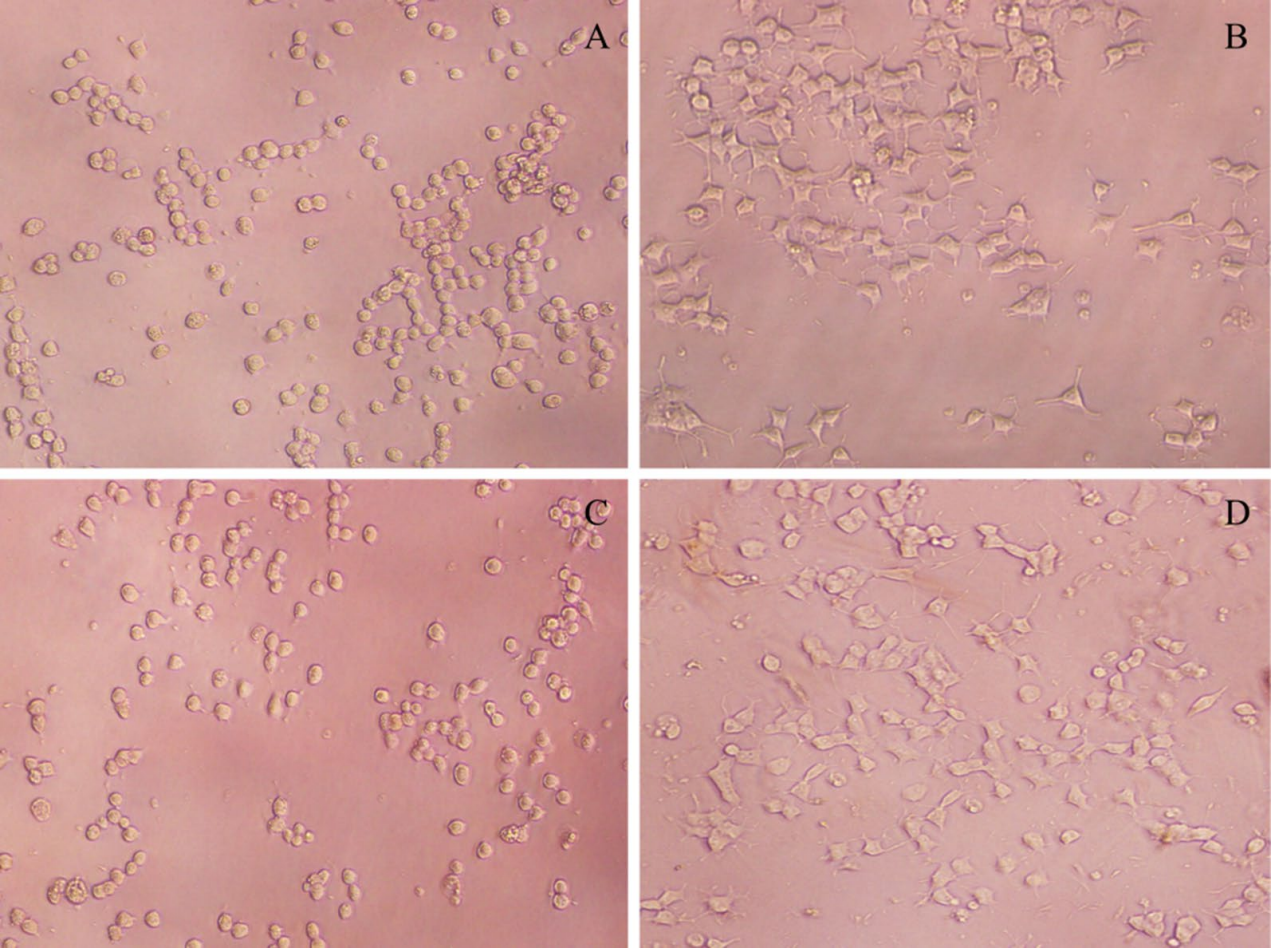
Protective effects of compound **VA-06** against CoCl_2_-induced injury in differentiated PC12 cells (×200) The most representative fields are shown. **A** Undifferentiated PC12 cells. **B** Differentiated PC12 cells by NGF. **C** CoCl_2_-induced neurotoxicity of differentiated PC12 cells. **D** CoCl_2_-induced neurotoxicity +**VA-06** (60 μM)

**Fig. 3:**
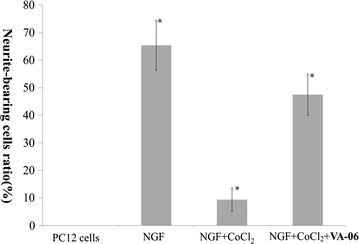
Protective effects of compound **VA-06** (60 μM) against CoCl_2_-induced injury in differentiated PC12 cells The neurite-bearing ration was shown as mean ± SD of at least 3 independent experiments. *p ≤ 0.05 level, significance relative to CoCl_2_ group

To further characterize the protective effect of **VA-06** on injured PC12 cells, the cell morphology changes were observed under an optical microscopy. As shown in Fig. 2, the morphology of undifferentiated PC12 cells was normal, the cells were small and proliferated to form clone-like cell clusters without neural characteristics (Fig. 2A); By exposure to NGF, normal differentiated PC12 cells showed round cell bodies with fine dendritic networks similar to those nerve cells (Fig. 2B). Moreover, the mean value expressed as percent of neurite-bearing cells in NGF treated cells was 65.4% (Fig. 3). When the differentiated PC12 cells treated with 250 mM CoCl_2_ for 12 h, almost all cells showed typical morphological changes such as cell body shrinkage and the disruption of the dendritic networks (Fig. 2C); the mean value of neurite-bearing cells (9.4%, Fig. 3) showed a significant decrease. While pretreatment with 60 μM **VA-06** before delivery of CoCl_2_ dramatically alleviated the damage caused by CoCl_2_ to cell morphology (Fig. 2D) and showed significant difference in the number of neurite-bearing cells (47.5%, Fig. 3) from that of CoCl_2_ treatment alone.

## Conclusions

In this study, we successfully synthesized 20 novel **T-VA** amide derivatives by combining **T-VA** with different amines. Their protective effects against CoCl_2_-induced neurotoxicity in differentiated PC12 cells were determined by the MTT assay. The result indicated that most of **T-VA** amide derivatives showed protective effects on injured differentiated PC12 cells. Among them, a large portion of the derivatives were more active (with lower EC_50_ values) than the positive control drug **TMP**, of which compound **VA-06** displayed the highest neuroprotective effect with EC_50_ values of 17.39 ± 1.34 µM. Although none of the newly synthesized T-VA derivatives showed more effect than the positive control drug **T-VA**, the results enriched the study of ligustrazine derivatives with neuroprotective activity. Further bioassay of compound **VA-06** on neuroprotective activity on animal models is underway.

## Methods

### Chemistry

Reagents were bought from commercial suppliers without any further purification. Melting points were measured at a rate of 5 °C/min using an X-5 micro melting point apparatus (Beijing, China) and were not corrected. Reactions were monitored by TLC using silica gel coated aluminum sheets (Qingdao Haiyang Chemical Co., Qingdao, China). NMR spectra were recorded on a BRUKER AVANCE 500 NMR spectrometer (Fällanden, Switzerland) with tetramethylsilane (TMS) as an internal standard; chemical shifts δ were given in ppm and coupling constants J in Hz. HR-MS were acquired using a Thermo Sientific TM LTQ Orbitrap XL hybrid FTMS instrument (Thermo Technologies, New York, NY, USA). Cellular morphologies were observed using an inverted fluorescence microscope (Olympus IX71, Tokyo, Japan).

#### Synthesis of (3,5,6-trimethylpyrazin-2-yl)methanol (**1**)

Compound **1** was prepared according to our previously reported method [21].

#### Synthesis of (3,5,6-trimethylpyrazin-2-yl)methyl 4-methylbenzenesulfonate (**2**)

To a solution of compound **1** (7.0 g, 46.3 mmol) and KOH (2.6 g, 46.3 mmol) in dry THF (100 ml), Tscl (8.82 g, 46.3 mmol) was added, then the mixture was stirred at 25 °C for 15 h. After completion of the reaction (as monitored by TLC), the reaction mixture was poured into water and the crude product was extracted with dichloromethane (3 × 100 ml), the combined organic layers were washed with brine (100 ml), anhydrous Na_2_SO_4_, filtered and the solvents were evaporated under vacuum. The crude products were purified by flash chromatography (Petroleum ether:Ethyl acetate = 4:1) to produce a white solid. The crude product, with 90% purity, was not purified further.

#### Synthesis of methyl 4-hydroxy-3-methoxybenzoate (**3**)

To a solution of vanillic acid (5.502 g, 32.7 mmol) in dry MeOH (100 ml), 3 ml SOCl_2_ was added gradually with stirring and cooling. Upon completion of the addition, the mixture was stirred at 25 °C for 15 h. After completion of the reaction (as monitored by TLC), the reaction mixture was evaporated under vacuum to produce a white solid. The crude product, with 95% purity, was not purified further.

#### Synthesis of methyl 3-methoxy-4-[(3,5,6-trimethylpyrazin-2-yl)methoxy] benzoate (**VA-01**)

Compound **2** (7.828 g, 256 mmol) and Compound **3** (3.580 g, 197 mmol) were dissolved in dry DMF, then K_2_CO_3_ (5.423 g, 393 mmol) was added and the mixture was kept at 70 °C for 15 h under nitrogen atmosphere. After completion of the reaction (as monitored by TLC), the reaction mixture was poured into ice-water and the crude product was extracted with dichloromethane. After drying the organic layer over anhydrous Na_2_SO_4_ and evaporating the solvent under vacuum, the crude products were purified by flash chromatography (Dichloromethane: methyl alcohol = 40:1) to produce a white solid.

##### methyl 3-methoxy-4-[(3,5,6-trimethylpyrazin-2-yl)methoxy] benzoate (**VA-01**)

White solid, yield: 52.5%, m.p.: 140.0–140.7 °C. ^1^H-NMR (CDCl_3_) (ppm): 2.51 (s, 3H, –CH_3_), 2.52 (s, 3H, –CH_3_), 2.62 (s, 3H, –CH_3_), 3.88 (s, 6H, 2× –OCH_3_), 5.26 (s, 2H, –CH_2_), 7.06 (d, J = 8.4 Hz, 1H, Ar–H), 7.53 (d, J = 1.2 Hz, 1H, Ar–H), 7.63 (dd, J = 1.2, 8.4 Hz, 1H, Ar–H). ^13^C-NMR (CDCl_3_) (ppm): 20.67 (–CH_3_), 21.51 (–CH_3_), 21.70 (–CH_3_), 52.16 (–OCH_3_), 56.12 (–OCH_3_), 70.81 (–CH_2_), 112.51, 112.82, 114.38, 123.41, 145.41, 148.91, 149.30, 150.12, 151.39, 151.99, 166.95 (–COO–). HRMS (ESI) m/z: 317.14905–3.4 ppm [M+H]^+^, calcd. for C_17_H_20_N_2_O_4_ 316.14231.

#### Synthesis of 3-Methoxy-4-[(3,5,6-trimethylpyrazin-2-yl)methoxy]benzoic acid (**VA-02**)

An aqueous solution of LiOH (1.289 g, 307 mmol) was added to a solution of **VA-01** (3.237 g, 102 mmol) in THF:MeOH:H_2_O = 3:1:1 (100 ml). The mixture was stirred at 37 °C for 2 h (checked by TLC). Upon completion of the reaction, pH was adjusted to 4–5 with 1 mol/l HCl. Then the reaction mixture was filtered and washed with water to give a white solid. The compound **VA-02** has been reported by us previously [20].

#### General procedure for the preparation of ligustrazine-vanillic acid derivative **VA-03**–**VA-20**

Compound **VA-02** (0.662 mmol, 1.0 eq) and the corresponding amine (0.926 mmol, 1.4 eq) were dissolved in 25 ml dry CH_2_Cl_2_, then HoBt (1.0592 mmol, 1.6 eq), EDCI (1.0592 mmol, 1.6 eq), DIPEA (1.986 mmol, 3.0 eq) were added and the mixture was kept at 25 °C for 12 h. After completion of the reaction (as monitored by TLC), the reaction mixture was poured into water and the crude product was extracted with dichloromethane (3 × 25 ml), the combined organic layers were washed with brine (50 ml), anhydrous Na_2_SO_4_, filtered and the solvents were evaporated under vacuum. The crude products were purified by flash chromatography (Petroleum ether:acetone = 5:1).

##### N-ethyl-3-methoxy-4-((3,5,6-trimethylpyrazin-2-yl)methoxy)benzamide (**VA-03**)

White solid, yield: 89.5%, m.p.: 194.5–195.8 °C. ^1^H-NMR (CDCl_3_) (ppm): 1.22 (t, 3H, –CH_3_), 2.49 (s, 3H, –CH_3_), 2.50 (s, 3H, –CH_3_), 2.60 (s, 3H, –CH_3_), 3.45 (m, 2H, –CH_2_), 3.86 (s, 3H, –OCH_3_), 5.22 (s, 2H, –CH_2_), 6.15 (s, 1H, –NH), 7.01 (d, J = 8.3 Hz, 1H, Ar–H), 7.21 (d, J = 8.3 Hz, 1H, Ar–H), 7.40 (s, 1H, Ar–H). ^13^C-NMR (CDCl_3_) (ppm): 15.06 (–CH_3_), 20.65 (–CH_3_), 21.48 (–CH_3_), 21.68 (–CH_3_), 35.03 (–CH_2_), 56.11 (–OCH_3_), 70.89 (–CH_2_), 111.12, 113.09, 118.99, 128.30, 145.49, 148.81, 149.73, 150.13, 150.55, 151.33, 167.04 (–CONH–). HRMS (ESI) m/z: 330.18045–3.9 ppm [M+H]^+^, calcd. for C_18_H_23_N_3_O_3_ 329.17394.

##### (3-methoxy-4-((3,5,6-trimethylpyrazin-2-yl)methoxy)phenyl)(piperidin-1-yl)methanone (**VA-04**)

White solid, yield: 65.2%, m.p.: 176.0–176.8 °C. ^1^H-NMR (CDCl_3_) (ppm): 1.66 (m, 6H, 3× –CH_2_), 2.50 (s, 3H, –CH_3_), 2.51 (s, 3H, –CH_3_), 2.61 (s, 3H, –CH_3_), 3.39 (brs, 2H, –CH_2_), 3.70 (m, 2H, –CH_2_), 3.84 (s, 3H, –OCH_3_) 5.21 (s, 2H, –CH_2_), 6.90 (d, J = 8.1 Hz, 1H, Ar–H), 6.96 (s, 1H, Ar–H), 7.01 (d, J = 8.1 Hz, 1H, Ar–H), ^13^C-NMR (CDCl_3_) (ppm): 20.70 (–CH_3_), 21.51 (–CH_3_), 21.73 (–CH_3_), 24.73, 31.11, 56.03 (–OCH_3_), 58.48, 71.00 (–CH_2_), 111.06, 113.45, 119.61, 129.68, 145.62, 148.75, 148.92, 149.65, 150.20, 151.30, 170.21 (–CON–). HRMS (ESI) m/z: 370.21179–3.4 ppm [M+H]^+^, calcd. for C_21_H_27_N_3_O_3_ 369.20524.

##### 3-methoxy-N-methyl-4-((3,5,6-trimethylpyrazin-2-yl)methoxy)benzamide (**VA-05**)

White solid, yield: 87.0%, m.p.:173.5–174.5 °C. ^1^H-NMR (CDCl_3_) (ppm): 2.50 (s, 3H, –CH_3_), 2.51 (s, 3H, –CH_3_), 2.61 (s, 3H, –CH_3_), 2.98 (s, 3H, –CH_3_), 3.86 (s, 3H, –OCH_3_), 5.23 (s, 2H, –CH_2_), 6.20 (s, 1H, –NH), 7.02 (d, J = 8.0 Hz, 1H, Ar–H), 7.21 (d, J = 8.0 Hz, 1H, Ar–H), 7.40 (s, 1H, Ar–H). ^13^C-NMR (CDCl_3_) (ppm): 20.68 (–CH_3_), 21.49 (–CH_3_), 21.71 (–CH_3_), 26.97 (–CH_3_), 56.11 (–OCH_3_), 70.90 (–CH_2_), 111.08, 113.12, 119.06, 128.16, 145.48, 148.83, 149.73, 150.15, 150.60, 151.37, 167.87 (–CONH–). HRMS (ESI) m/z: 316.16489–3.9 ppm [M+H]^+^, calcd. for C_17_H_21_N_3_O_3_ 315.15829.

##### N-(3-(dimethylamino)phenyl)-3-methoxy-4-((3,5,6-trimethylpyrazin-2-yl)methoxy)benzamide (**VA-06**)

White solid, yield: 74.0%, m.p.: 171.4–172.3°C. ^1^H-NMR (CDCl_3_) (ppm): 2.51 (s, 6H, 2× –CH_3_), 2.62 (s, 3H, –CH_3_), 2.98 (s, 6H, 2× –CH_3_), 3.91 (s, 3H, –OCH_3_), 5.27 (s, 2H, –CH_2_), 6.53 (d, J = 7.8 Hz, 1H, Ar–H), 6.81 (d, J = 7.8 Hz, 1H, Ar–H), 7.09 (d, J = 8.4 Hz, 1H, Ar–H), 7.20 (m, 1H, Ar–H), 7.33 (dd, J = 1.9 Hz, 8.4 Hz, 1H, Ar–H), 7.51 (d, J = 1.9 Hz, 1H, Ar–H), 7.69 (s, 1H, –NH). ^13^C-NMR (CDCl_3_) (ppm): 20.70 (–CH_3_), 21.53 (–CH_3_), 21.74 (–CH_3_), 41.1 (–CH_3_), 56.10 (–OCH_3_), 70.74 (–CH_2_), 103.80, 109.96, 111.25,111.40, 119.51, 120.83, 128.70, 129.82, 137.45, 145.34, 148.91, 149.22, 150.14, 151.45, 151.94, 152.52, 166.97 (–CON–). HRMS (ESI) m/z: 421.22144–6.0 ppm [M+H]^+^, calcd. for C_24_H_28_N_4_O_3_ 420.21614.

##### 3-methoxy-N-(3-(2-methyl-1H-imidazol-1-yl)propyl)-4-((3,5,6-trimethylpyrazin-2-yl)methoxy)benzamide (**VA-07**)

White solid, yield: 68.9%, m.p.: 160.0–160.8 °C. ^1^H-NMR (CDCl_3_) (ppm): 2.04 (m, 2H, –CH_2_), 2.35 (s, 3H, –CH_3_), 2.48 (s, 3H, –CH_3_), 2.49 (s, 3H, –CH_3_), 2.59 (s, 3H, –CH_3_), 3.45 (m, 2H, –CH_2_), 3.86 (s, 3H, –OCH_3_), 3.93 (m, 2H, –CH_2_), 5.21 (s, 2H, –CH_2_), 6.66 (m, 1H, –NH), 6.90 (s, 2H, 2× –CH), 7.02 (d, J = 8.4 Hz, 1H, Ar–H), 7.23 (d, J = 8.4 Hz, 1H, Ar–H), 7.40 (s, 1H, Ar–H). ^13^C-NMR (CDCl_3_) (ppm): 12.98 (–CH_3_), 20.78 (–CH_3_), 21.50 (–CH_3_), 21.83 (–CH_3_), 30.89 (–CH_2_), 37.46 (–CH_2_), 44.19 (–CH_2_), 56.16 (–OCH_3_), 70.91 (–CH_2_), 111.08, 113.01, 119.37, 119.44, 126.73, 127.48, 144.46, 145.24, 148.70, 149.71, 150.24, 150.88, 151.55, 167.45 (–CONH–). HRMS (ESI) m/z: 424.23187–7.1 ppm [M+H]^+^, calcd. for C_23_H_29_N_5_O_3_ 423.22704.

##### N-(3-ethoxypropyl)-3-methoxy-4-((3,5,6-trimethylpyrazin-2-yl)methoxy)benzamide (**VA-08**)

White solid, yield: 76.4%, m.p.: 119.0–119.9 °C. ^1^H-NMR (CDCl_3_) (ppm): 1.23 (m, 3H, –CH_3_), 1.88 (m, 2H, –CH_2_), 2.50 (s, 3H, –CH_3_), 2.51 (s, 3H, –CH_3_), 2.61 (s, 3H, –CH_3_), 3.50 (m, 2H, –CH_2_), 3.55 (m, 2H, –CH_2_), 3.61 (m, 2H, –CH_2_), 3.88 (s, 3H, –OCH_3_), 5.24 (s, 2H, –CH_2_–), 7.03 (d, J = 8.3 Hz, 1H, Ar–H), 7.07 (s, 1H, –NH), 7.20 (d, J = 8.3 Hz, 1H, Ar–H), 7.42 (s, 1H, Ar–H). ^13^C-NMR (CDCl_3_) (ppm): 15.52 (–CH_3_), 20.75 (–CH_3_), 21.51 (–CH_3_), 21.78 (–CH_3_), 28.88 (–CH_2_), 39.70, 56.11 (–OCH_3_), 58.58, 66.73, 70.83 (–CH_2_), 111.05, 112.97, 118.94, 128.32, 145.46, 148.75, 149.65, 150.24, 150.46, 151.41, 166.80 (–CONH–). HRMS (ESI) m/z: 388.22171–5.0 ppm [M+H]^+^, calcd. for C_21_H_29_N_3_O_4_ 387.21581.

##### N-(2-hydroxyethyl)-3-methoxy-4-((3,5,6-trimethylpyrazin-2-yl)methoxy)benzamide (**VA-09**)

Brick-red solid, yield: 86.7%, m.p.: 156.9–157.9 °C. ^1^H-NMR (CDCl_3_) (ppm): 2.50 (s, 3H, –CH_3_), 2.51 (s, 3H, –CH_3_), 2.61 (s, 3H, –CH_3_), 3.59 (m, 2H, –CH_2_), 3.81 (m, 2H, –CH_2_), 3.87 (s, 3H, –OCH_3_), 5.23 (s, 2H, –CH_2_), 6.63 (s, 1H, –NH), 7.03 (d, J = 8.4 Hz, 1H, Ar–H), 7.25 (dd, J = 2.0, 8.4 Hz, 1H, Ar–H), 7.40 (d, J = 2.0 Hz, 1H, Ar–H). ^13^C-NMR (CDCl_3_) (ppm): 20.65 (–CH_3_), 21.42 (–CH_3_), 21.69 (–CH_3_), 43.01 (–CH_2_), 56.08 (–OCH_3_), 62.27 (–CH_2_), 70.71 (–CH_2_), 111.07, 112.97, 119.50, 127.54, 145.25, 148.83, 149.61, 150.16, 150.80, 151.54, 168.15 (–CONH–). HRMS (ESI) m/z: 346.17517–4.4 ppm [M+H]^+^, calcd. for C_18_H_23_N_3_O_4_ 345.16886.

##### N-(2-(dimethylamino)ethyl)-3-methoxy-4-((3,5,6-trimethylpyrazin-2-yl)methoxy)benzamide (**VA-10**)

White solid, yield: 79.3%, m.p.: 148.6–149.0 °C. ^1^H-NMR (CDCl_3_) (ppm): 2.51 (s, 6H, 2× –CH_3_), 2.52 (s, 2H, –CH_2_), 2.54 (s, 6H, 2× –CH_3_), 2.62 (s, 3H, –CH_3_), 3.92 (s, 3H, –OCH_3_), 4.65 (d, 2H, –CH_2_), 5.26 (s, 2H, –CH_2_–), 7.09 (d, J = 8.4 Hz, 1H, Ar–H), 7.38 (dd, J = 2.0, 8.4 Hz, 1H, Ar–H), 7.51 (d, J = 2.0 Hz, 1H, Ar–H), 7.82 (brs, 1H, –NH). ^13^C-NMR (CDCl_3_) (ppm): 20.75 (–CH_3_), 21.48 (–CH_3_), 21.79 (–CH_3_), 27.41, 32.33, 51.08, 56.14 (–OCH_3_), 70.92 (–CH_2_), 111.35, 113.07, 118.72, 128.48, 145.34, 148.68, 149.82, 150.24, 150.64, 151.49, 167.32 (–CONH–). HRMS (ESI) m/z: 373.23010+16.4 ppm [M+H]^+^, calcd. for C_20_H_28_N_4_O_3_ 372.21614.

##### (4-(4-chlorophenyl)piperazin-1-yl)(3-methoxy-4-((3,5,6-trimethylpyrazin-2-yl)methoxy)phenyl)methanone (**VA-11**)

White solid, yield: 68.3%, m.p.: 179.0–179.5 °C. ^1^H-NMR (CDCl_3_) (ppm): 2.51 (s, 3H, –CH_3_), 2.53 (s, 3H, –CH_3_), 2.63 (s, 3H, –CH_3_), 3.16 (brs, 4H, 2× –CH_2_), 3.79 (brs, 4H, 2× –CH_2_), 3.86 (s, 3H, –OCH_3_), 5.24 (s, 2H, –CH_2_), 6.87 (d, J = 8.2 Hz, 2H, Ar–H), 6.96 (d, J = 8.2 Hz, 1H, Ar–H), 7.01 (s, 1H, Ar–H), 7.05 (d, J = 8.2 Hz, 1H, Ar–H), 7.23 (d, J = 8.2 Hz, 2H, Ar–H). ^13^C-NMR (CDCl_3_) (ppm): 20.62 (–CH_3_), 21.51 (–CH_3_), 21.65 (–CH_3_), 29.83, 32.08, 37.07, 49.99 (–CH_2_), 56.15 (–OCH_3_), 71.04 (–CH_2_), 111.46, 113.53, 118.14, 120.08, 128.59, 129.30, 145.67, 148.90, 149.48, 149.90, 150.13, 151.29, 170.37 (–CON–). HRMS (ESI) m/z: 481.19775–6.0 ppm [M+H]^+^, calcd. for C_26_H_29_ClN_4_O_3_ 480.19282.

##### tert-butyl4-(3-methoxy-4-((3,5,6-trimethylpyrazin-2-yl)methoxy)benzoyl)piperazine-1-carboxylate (**VA-12**)

White solid, yield: 57.6%, m.p.: 86.6–87.6 °C. ^1^H-NMR (CDCl_3_) (ppm): 1.36 (brs, 2H, –CH_2_), 1.44 (s, 9H, 3× –CH_3_), 1.99 (brs, 2H, –CH_2_), 2.50 (s, 3H, –CH_3_), 2.52 (s, 3H, –CH_3_), 2.62 (s, 3H, –CH_3_), 3.02 (brs, 2H, –CH_2_), 3.70 (brs, 2H, –CH_2_), 3.84 (s, 3H, –OCH_3_), 4.47 (brs, 2H, –CH_2_), 5.22 (s, 2H, –CH_2_–), 6.90 (dd, J = 1.6 Hz, 8.2 Hz, 1H, Ar–H), 6.96 (d, J = 1.6 Hz, 1H, Ar–H), 7.02 (d, J = 8.2 Hz, 1H, Ar–H). ^13^C-NMR (CDCl_3_) (ppm): 20.64 (–CH_3_), 21.49 (–CH_3_), 21.66 (–CH_3_), 28.49 (–CH_3_), 33.01, 41.35, 48.08 (–CH), 56.09 (–OCH_3_), 71.03 (–CH_2_), 79.75 (–OCH), 111.22, 113.55, 119.77, 129.10, 145.66, 148.83, 149.26, 149.79, 150.14, 151.26, 155.16 (–COO–), 170.35 (–CON–). HRMS (ESI) m/z: 485.27286–7.3 ppm [M+H]^+^, calcd. for C_26_H_36_N_4_O_5_ 484.26857.

##### N-(4-(cyanomethyl)phenyl)-3-methoxy-4-((3,5,6-trimethylpyrazin-2-yl)methoxy)benzamide (**VA-13**)

White solid, yield: 65.7%, m.p.:199.0–199.5 °C. ^1^H-NMR (CDCl_3_) (ppm): 2.51 (s, 3H, –CH_3_), 2.52 (s, 3H, –CH_3_), 2.62 (s, 3H, –CH_3_), 3.74 (s, 2H, –CH_2_), 3.90 (s, 3H, –OCH_3_), 5.27 (s, 2H, –CH_2_), 7.09 (d, J = 8.2 Hz, 1H, Ar–H), 7.32 (d, 2H, Ar–H) 7.35 (dd, J = 1.8, 8.2 Hz, 1H, Ar–H), 7.48 (s, 1H, Ar–H), 7.65 (d, J = 8.2 Hz, 2H, Ar–H), 7.87 (brs, 1H, –NH). ^13^C-NMR (CDCl_3_) (ppm): 20.66 (–CH_3_), 21.47 (–CH_3_), 21.70 (–CH_3_), 23.24, 56.14 (–OCH_3_), 70.80 (–CH_2_), 111.24, 112.96, 118.09, 119.51, 120.83, 125.59, 127.96, 128.70, 138.15, 145.27, 148.92, 149.85, 150.11, 151.16, 151.51, 165.45 (–CON–). HRMS (ESI) m/z: 417.19052–5.2 ppm [M+H]^+^, calcd. for C_24_H_24_N_4_O_3_ 416.18484.

##### 3-methoxy-N-(4-phenoxyphenyl)-4-((3,5,6-trimethylpyrazin-2-yl)methoxy)benzamide (**VA-14**)

White solid, yield: 57.8%, m.p.: 182.5–183.3 °C. ^1^H-NMR (CDCl_3_) (ppm): 2.52 (s, 3H, –CH_3_), 2.53 (s, 3H, –CH_3_), 2.64 (s, 3H, –CH_3_), 3.91 (s, 3H, –OCH_3_), 5.27 (s, 2H, –CH_2_), 7.01 (m, 4H, Ar–H), 7.09 (m, 2H, Ar–H), 7.33 (m, 3H, Ar–H), 7.49 (d, J = 2 Hz, 1H, Ar–H), 7.58 (m, 2H, Ar–H), 7.78 (brs, 1H, –NH). ^13^C-NMR (CDCl_3_) (ppm): 20.63 (–CH_3_), 21.50 (–CH_3_), 21.66 (–CH_3_), 56.16 (–OCH_3_), 70.85 (–CH_2_), 111.27, 113.07, 118.59, 120.04, 119.75, 122.04, 123.23, 128.25, 129.86, 133.66, 145.40, 148.96, 149.90, 150.09, 151.03, 151.42, 153.68, 157.62, 165.35 (–CON–). HRMS (ESI) m/z: 470.20447–7.5 ppm [M+H]^+^, calcd. for C_28_H_27_N_3_O_4_ 469.20016.

##### 3-methoxy-N-phenyl-4-((3,5,6-trimethylpyrazin-2-yl)methoxy)benzamide (**VA-15**)

White solid, yield: 68.9%, m.p.: 189.7–190.2 °C. ^1^H-NMR (CDCl_3_) (ppm): 2.50 (s, 3H, –CH_3_), 2.51 (s, 3H, –CH_3_), 2.62 (s, 3H, –CH_3_), 3.89 (s, 3H, –OCH_3_), 5.26 (s, 2H, –CH_2_–), 7.08 (d, J = 8.3 Hz, 1H, Ar–H), 7.14 (m, 1H, Ar–H), 7.35 (m, 3H, Ar–H), 7.49 (d, J = 1.8 Hz, 1H, Ar–H), 7.62 (d, 2H, Ar–H), 7.81 (s, 1H, –NH–). ^13^C-NMR (CDCl_3_) (ppm): 20.65 (–CH_3_), 21.47 (–CH_3_), 21.69 (–CH_3_), 56.08 (–OCH_3_), 70.81 (–CH_2_), 111.25, 112.95, 119.39, 120.26, 124.46, 128.33, 129.12, 138.19, 145.29, 148.87, 149.81, 150.10, 150.99, 151.46, 165.42 (–CONH–). HRMS (ESI) m/z: 378.18002–4.6 ppm [M+H]^+^, calcd. for C_22_H_23_N_3_O_3_ 377.17394.

##### 3-methoxy-N-(naphthalen-2-yl)-4-((3,5,6-trimethylpyrazin-2-yl)methoxy)benzamide (**VA-16**)

White solid, yield: 67.0%, m.p.: 174.1–175.0 °C.^1^H-NMR (CDCl_3_) (ppm): 2.53 (s, 6H, 2× –CH_3_), 2.65 (s, 3H, –CH_3_), 3.92 (s, 3H, –OCH_3_), 5.30 (s, 2H, –CH_2_), 7.14 (d, J = 8.2 Hz, 1H, Ar–H), 7.52 (m, 4H, Ar–H), 7.58 (s, 1H, Ar–H), 7.74 (d, J = 8.2 Hz, 1H, Ar–H), 7.90 (m, 2H, Ar–H), 7.99 (m, 1H, Ar–H), 8.17 (s, 1H, –NH–). ^13^C-NMR (CDCl_3_) (ppm): 20.66 (–CH_3_), 21.49 (–CH_3_), 21.66 (–CH_3_), 56.16 (–OCH_3_), 70.86 (–CH_2_), 111.49, 113.05, 119.44, 121.03, 121.47, 125.88, 126.15, 126.43, 127.73, 128.19, 128.87, 132.70, 134.25, 145.39, 148.93, 149.94, 150.11, 151.11, 151.43, 166.02 (–CONH–). HRMS (ESI) m/z: 428.19547–4.6 ppm [M+H]^+^, calcd. for C_26_H_25_N_3_O_3_ 427.18959.

##### 3-methoxy-N-(3-morpholinopropyl)-4-((3,5,6-trimethylpyrazin-2-yl)methoxy)benzamide (**VA-17**)

White solid, yield: 65.2%, m.p.: 129.2–129.5 °C. ^1^H-NMR (CDCl_3_) (ppm): 1.79 (m, 2H, –CH_2_), 2.50 (m, 10H), 2.55 (m, 2H, –CH_2_), 2.61 (s, 3H, –CH_3_), 3.55 (m, 2H, –CH_2_), 3.70 (m, 4H, 2× –CH_2_), 3.89 (s, 3H, –OCH_3_), 5.25 (s, 2H, –CH_2_), 7.05 (d, J = 8.3 Hz, 1H, Ar–H), 7.24 (dd, J = 1.6, 8.3 Hz, 1H, Ar–H), 7.47 (d, J = 1.6 Hz, 1H, Ar–H), 7.75 (brs, 1H, –NH–). ^13^C-NMR (CDCl_3_) (ppm): 20.79 (–CH_3_), 21.47 (–CH_3_), 21.82 (–CH_3_), 24.40, 40.42 (–CH_2_), 53.86 (–CH_2_), 56.19 (–OCH_3_), 58.59, 66.90, 70.91 (–CH_2_), 111.42, 112.94, 118.95, 128.28, 145.34, 148.67, 149.77, 150.26, 150.59, 151.47, 167.06 (–CONH–). HRMS (ESI) m/z: 429.24731–6.6 ppm [M+H]^+^, calcd. for C_23_H_32_N_4_O_4_ 428.24232.

##### 3-methoxy-N-(thiophen-2-ylmethyl)-4-((3,5,6-trimethylpyrazin-2-yl)methoxy)benzamide (**VA-18**)

White solid, yield: 62.7%, m.p.:156.3–156.9 °C. ^1^H-NMR (CDCl_3_) (ppm): 2.50 (s, 3H, –CH_3_), 2.52 (s, 3H, –CH_3_), 2.62 (s, 3H, –CH_3_), 3.89 (s, 3H, –OCH_3_), 4.80 (d, 2H, –CH_2_), 5.24 (s, 2H, –CH_2_), 6.36 (brs, 1H, –NH), 6.97 (m, 1H, –CH), 7.03 (m, 2H, 2× –CH), 7.22 (dd, J = 2.0, 8.3 Hz, 1H, Ar–H), 7.24 (d, 1H, Ar–H), 7.44 (d, J = 2.0 Hz, 1H, Ar–H). ^13^C-NMR (CDCl_3_) (ppm): 20.42 (–CH_3_), 21.47 (–CH_3_), 29.84 (–CH_3_), 38.97 (–CH_2_), 56.18 (–OCH_3_), 70.80 (–CH_2_), 111.28, 113.13, 119.22, 125.50, 126.36, 127.09, 127.66, 141.03, 144.09, 145.78, 149.19, 149.83, 150.80, 151.46, 166.73 (–CONH–). HRMS (ESI) m/z: 398.15253–3.3 ppm [M+H]^+^, calcd. for C_21_H_23_N_3_O_3_ S 397.14601.

##### 3-methoxy-N-(4-methoxybenzyl)-4-((3,5,6-trimethylpyrazin-2-yl)methoxy)benzamide (**VA-19**)

White solid, yield: 75.1%, m.p.: 161.6–162.3 °C. ^1^H-NMR (CDCl_3_) (ppm): 2.48 (s, 3H, –CH_3_), 2.49 (s, 3H, –CH_3_), 2.59 (s, 3H, –CH_3_), 3.78 (s, 3H, –OCH_3_), 3.86 (s, 3H, –OCH_3_), 4.53 (d, 2H, –CH_2_), 5.22 (s, 2H, –CH_2_), 6.41 (s, 1H, –NH), 6.85 (s, 1 H, Ar–H), 6.86 (d, J = 8.0 Hz, 2 H, Ar–H), 7.00 (d, J = 8.3 Hz, 1 H, Ar–H), 7.19 (m, 1 H, Ar–H),, 7.25 (d, J = 8.0 Hz, 2 H, Ar–H), 7.43 (s, 1H, Ar–H). ^13^C-NMR (CDCl_3_) (ppm): 20.68 (–CH_3_), 21.50 (–CH_3_), 21.72 (–CH_3_), 43.72 (–CH_2_–), 55.2 (–OCH_3_), 56.10 (–OCH_3_), 70.81 (–CH_2_), 111.12, 112.92, 114.17, 119.11, 127.79, 129.42, 130.44, 145.38, 148.79, 149.68, 150.15, 150.67, 151.41, 159.13, 166.87 (–CONH–). HRMS (ESI) m/z: 422.21408–14.0 ppm [M+H]^+^, calcd. for C_24_H_27_N_3_O_4_ 421.20016.

##### Methyl 3-(3-methoxy-4-((3,5,6-trimethylpyrazin-2-yl)methoxy)benzamido)propanoate (**VA-20**)

White solid, yield: 83.2%, m.p.: 139.6–140.1 °C. ^1^H-NMR (CDCl_3_) (ppm): 2.51 (s, 3H, –CH_3_), 2.52 (s, 3H, –CH_3_), 2.61 (s, 3H, –CH_3_), 2.64 (t, 2H, –CH_2_), 3.69 (m, 2H, –CH_2_), 3.70 (s, 3H, –OCH_3_), 3.88 (s, 3H, –OCH_3_), 5.24 (s, 2H, –CH_2_), 6.80 (s, 1H, –NH), 7.02 (d, J = 8.3 Hz, 1H, Ar–H), 7.20 (d, J = 8.3 Hz, 1H, Ar–H), 7.40 (s, 1H, Ar–H). ^13^C-NMR (CDCl_3_) (ppm): 20.59 (–CH_3_), 21.52 (–CH_3_), 21.63 (–CH_3_), 33.82 (–CH_2_), 35.36 (–CH_2_), 52.02 (–OCH_3_), 56.12 (–OCH_3_), 70.80 (–CH_2_), 111.06, 112.97, 119.15, 127.75, 145.56, 147.42, 149.67, 150.06, 150.66, 151.30, 166.97 (–CONH–), 173.61 (–COO–). HRMS (ESI) m/z: 388.18057–17 ppm [M+H]+, calcd. for C_20_H_25_N_3_O_5_ 387.17942.

## Bio-evaluation methods

### Cell culture

PC12 cells were obtained from the Chinese Academy of Medical Sciences & Peking Union Medical College. The cultures of the PC12 cells were maintained as monolayer in RPMI 1640 supplemented with 10% (v/v) heat inactivated (Gibco) horse serum, 5% (v/v) fetal bovine serum and 1% (v/v) penicillin/streptomycin (Thermo Technologies, New York, NY,USA) and incubated at 37 °C in a humidified atmosphere with 5% CO_2_. **T-VA** amide derivatives were dissolved in dimethyl sulfoxide (DMSO).

### Protective effect on damaged differentiated pc12 cells

The neuroprotective effect of newly synthesized **T-VA** amide derivatives was evaluated in vitro via the MTT method on the differentiated PC12 cells damaged by CoCl_2_ with ligustrazine as the positive control. PC12 cells growing in the logarithmic phase were incubated in the culture dishe and allowed to grow to the desired confluence. Then the cells were switched to fresh serum-free medium and incubated for 14 h. At the end of this incubation, the PC12 cells were collected and resuspended in 1640 medium supplemented with 10% (v/v) fetal bovine serum, then the cells were seeded in poly-l-lysine-coated 96-well culture plates at a density of 7 × 10^3^ cells/well and incubated for another 48 h in the presence of 50 ng/ml NGF.

The differentiated PC12 cells were pretreated with serial dilutions of **T-VA** amide derivatives (60, 30, 15, 7.5, 3.75 µM) for 36 h, and then exposed to CoCl_2_ (final concentration, 250 mM) for another 12 h. Control differentiated cells were not treated with **T-VA** amide derivatives and CoCl_2_. At the end of this incubation, 20 μl of 5 mg/ml methylthiazol tetrazolium (MTT) was added to each well and incubation proceeded at 37 °C for another 4 h. After the supernatant medium was removed carefully, 200 μl dimethylsulphoxide (DMSO) were added to each well and absorbance was measured at 490 nm using a plate reader (BIORAD 550 spectrophotometer, Bio-rad Life Science Development Ltd., Beijing, China). The proliferation rates of damaged PC12 cells were calculated by the formula [OD_490_(Compd) − OD_490_(CoCl_2_)]/[OD_490_(NGF) − OD_490_(CoCl_2_)] × 100%; The concentration of the compounds which produces a 50% proliferation of surviving cells corresponds to the EC_50_. And it was calculated using the following equation: −pEC_50_ = log C_max_ − log 2 × (∑P − 0.75 + 0.25P_max_ + 0.25P_min_), where C_max_ = maximum concentration, ∑P = sum of proliferation rates, P_max_ = maximum value of proliferation rate and P_min_ = minimum value of proliferation rate [20–22].

### Observation of morphologic changes

The changes in cell morphology after treatment with **VA-06** were determined using light microscopy in this assay, it was performed as previously described [22]. Differentiation was scored as the cells with one or more growth cone tipped neurites greater than 2 cell bodies in length. The cell differentiation rate was calculated by the formula [the number of differentiated cells]/[the number of total cells] × 100%. Three fields were randomly chosen from different wells of three independent experiments. All data are expressed as mean ± standard deviation (SD). Statistical analyses were performed using SAS version 9.0 (SAS Institute Inc., Cary, NC, USA). Between-groups differences were assessed using Student t tests and p < 0.05 was considered significant.
